# ROR2-Related Skeletal Dysplasia Reveals Disrupted Chondrocyte Polarity through Modulation of BMP/TGF-β Signaling

**DOI:** 10.14336/AD.2023.0531

**Published:** 2024-02-01

**Authors:** Yichen Yao, Xin Wang, Lichieh Lin, Xiaolei Zhang, Yan Wang

**Affiliations:** ^1^Hospital of Stomatology, Guanghua School of Stomatology, Sun Yat-sen University, China.; ^2^Guangdong Provincial Key Laboratory of Stomatology, Guangzhou, China.; ^3^Department of Medicine, Harvard Medical School, Boston, MA, USA.; ^4^Jeff and Penny Vinik Center for Translational Immunology Research, Division of Allergy and Clinical Immunology, Brigham and Women’s Hospital, Boston, MA, USA.; ^5^Department of Stomatology, the Eighth Affiliated Hospital, Sun Yat-sen University, Shen Zhen, Guangdong, China.

**Keywords:** Robinow syndrome, Ror2, skeletal development, BMP/TGF-β signaling;

## Abstract

Genetic studies have shown that Robinow syndrome (RS), a rare skeletal dysplasia, is caused by ROR2 mutation. However, the cell origin and molecular mechanisms underlying this disease remain elusive. We established a conditional knockout system by crossing Prx1cre and Osxcre with Ror2 ^flox/flox^ mice. and conducted histological and immunofluorescence analyses to investigate the phenotypes during skeletal development. In the Prx1cre line, we observed RS-like skeletal abnormities, including short stature and an arched skull. Additionally, we found inhibition of chondrocyte differentiation and proliferation. In the Osxcre line, loss of ROR2 in osteoblast lineage cells led to reduced osteoblast differentiation during both embryonic and postnatal stages. Furthermore, ROR2 mutant mice exhibited increased adipogenesis in the bone marrow compared to their littermate controls. To further explore the underlying mechanisms, bulk RNA-seq analysis of Prx1cre; Ror2 ^flox/flox^ embryos was performed, results revealed decreased BMP/TGF-β signaling. Immunofluorescence analysis further confirmed the decreased expression of p-smad1/5/8, accompanied by disrupted cell polarity in the developing growth plate. Pharmacological treatment using FK506 partially rescued the skeletal dysplasia and resulted in increased mineralization and osteoblast differentiation. By modeling the phenotype of RS in mice, our findings provide evidence for the involvement of mesenchymal progenitors as the cell origin and highlight the molecular mechanism of BMP/TGF-β signaling in skeletal dysplasia.

## INTRODUCTION

Bone is one of the most vital human organs responsible for providing continuous support, facilitating movement, and ensuring protection [1]. Longitudinal skeletal growth through endochondral ossification at the growth plate. Dysregulation of signal transduction in this process can lead to phenotypes such as skeletal dysplasia [2, 3]. In vivo and in vitro studies have identified several paracrine pathways involved in chondrogenesis, includingfibroblast growth factor (FGF), bone morphogenetic proteins (BMPs), and hedgehog (HH) signaling [4, 5]. Robinow syndrome (RS) is an exceptionally rare genetic disorder characterized by short-limbed dwarfism, defects in vertebral segmentation, and abnormalities in the head, face, and external genitalia. It been found that RS is caused by mutations in Wnt/planar cell polarity (PCP)- related genes such as *WNT5A*, *ROR2*, *DVL1* and *DVL3* [6-8]. Understanding the cellular origin and molecular mechanisms underlying this rare disease can provide valuable insights into endochondral ossification and therapeutics targeting not only rare genetic diseases but also related common disorders.

Ror2 (A member of the Ror receptor tyrosine kinase family) is a transmembrane protein kinase that plays a crucial role in the non-canonical Wnt signaling pathway by mediating the activation of the PCP pathway [9]. The extracellular domain of Ror2 binds to the non-canonical Wnt/PCP signaling ligand Wnt5a and phosphorylates the tyrosine residues of intracellular target proteins to regulate cell polarity, proliferation, and differentiation [10]. During chondrogenesis, the PCP signal cascade is essential for the proximal to distal limb elongation in the developing murine growth plate [11]. Wnt5a is broadly expressed in the cartilage growth plate proliferative zone, pre-hypertrophic zone, and in the long bone, and the loss of Wnt5a in murine embryos results in abnormal limb morphology, delayed chondrocyte hypertrophy, and bone mineralization [12].

The PCP pathway is regulated by a group of highly conserved core proteins that asymmetrically localize at the cell surface throughout the polarized tissue via cell-to-cell interactions, providing directional information in the form of concentration gradients [13, 14]. Vangl2 is one of the core proteins of the PCP pathway, and its phosphorylation level regulates its activity [15]. Ror2 is required for the regulation of Vangl2 phosphorylation in the proximal-distal axis to establish chondrocyte PCP, and Ror2 knockout mice exhibit shortened limb buds [16, 17]. Although the PCP signaling pathway has become the primary focus for investigating the skeletal phenotype exhibited in rRS, the cellular origins and mechanisms by which Ror2 controls limb morphogenesis have yet to be determined. Further research is needed to elucidate the role of Ror2 and the PCP pathway in limb development and the pathogenesis of RS.

In this study, we investigated the role of Ror2 in limb morphogenesis and its connection to RS, a rare genetic disorder characterized by skeletal dysplasia. Givin that Prx1^+^ mesenchymal progenitor cells give rise to chondrocyte and osteoblast lineage cells [18], and Osx^+^ cells are the progeny of premature osteoblast [19]. . A conditional knockout system by crossing Prx1cre and Osxcre with Ror2 flox/flox mice were applied. We showed that Ror2 in mesenchymal progenitor cells that give rise to chondrocytes and osteoblasts, as well as in osteoprogenitor cells and hypertrophic chondrocytes in the mouse embryos. The mutants with Ror2 loss in early limb bud mesenchyme, but not in the osteoblast progenitors, showed short stature and arched skull with chondrodysplasia and skeletal dysplasia similar to symptoms observed in RS patients. We found that the ROR2 plays a crucial role in growth plate polarity during embryonic development, with dysregulated balance of osteogenic and audiogenic differentiation. We also found that ROR2 coordinates with BMP/TGF-β signaling, and activation of this signaling pathway might be a potential strategy to treat the skeletal defects of RS patients. Our study provides valuable insights into the cellular origins and molecular mechanisms underlying RS and related common disorders.

## MATERIAL AND METHODS

### Animals

Mice included in this study were purchased from the Jackson Laboratory (Prx1cre, stock no.005584, *Osx-GFP::cre*, stock no. 006361, *Ror2 ^flox/flox^*, stock no. 018354). All mice analyzed had a C57BL/6 background. Animals were maintained under specific pathogen-free conditions in the institutional animal facility of the Sun Yat-sen University. All animal studies were performed with a protocol approved by the Animal Ethical and Welfare Committee of Sun Yat-sen University (SYSU-IACUC- 2021001878).

### IF staining

Neonatal and postnatal tissues were fixed overnight at 4 °C in 4% paraformaldehyde (PFA) in phosphate-buffered saline (PBS) and then decalcified in 15% EDTA (VWR, BDH4616) for 4 weeks. Decalcified tissue was processed for either frozen or paraffin sections. Sections were rehydrated, permeabilized with PBST (1x PBS + 0.1% TritonX-100 (Sigma-Aldrich, T8787) for 15 min, blocked in 10% goat serum for 1 hour at room temperature and incubated in primary antibody overnight at 4 °C. Isotype antibody (Rabbit mAb IgG control, abcam, 172730) controls and secondary antibody-only controls were employed to validate antibody specificity and distinguish genuine target staining from the background. The following primary antibodies were used at a 1:400 dilution in PBS: rabbit polyclonal Osx (Abcam, ab22552), rabbit monoclonal Ki67 (Cell Signaling Technology, 9129), rabbit polyclonal Ocn (Abcam, ab93876), rabbit monoclonal Collagen X (Abcam, 260040), rabbit monoclonal SOX9 (Millipore, 5535), rabbit monoclonal p-smad1/5/8 (Cell Signaling Technology, 13820). Following the primary incubation, slides were washed in PBS for 30 min, incubated in a florescent secondary for 1 hour at room temperature, washed for 30 min, and mounted in media containing a nuclear stain (Abcam, 104139). The secondary antibody used was Alexa Fluor 568 goat anti-rabbit (Life Technologies, A11011, 1:500). Representative images were selected based on the mean values of fluorescent signals.

### Small molecule treatment

FK506 (MCE. Cas No. HY-13756) was prepared as described previously [20]. FK506 was injected to neonatal pups by intraperitoneal injection at a concentration of 1μM every other day from P0 to P7. Equivalent volumes of vehicles were injected to control animals.

### Histology

Bone tissues were fixed and decalcified as described above. For Safranin-O staining, sections were first submerged in Hematoxylin QS solution (Vector Laboratories. Inc. H3404) for 5 min, washed with running water for 5 min and rapidly destained in Acid EtOH. Slides were washed in running water for 2 min, stained with fast green solution for 5 min and quickly rinsed with 1% acetic acid solution for no more than 10-15 seconds. Then slides were stained with 0.1% safranin O solution for 5 min, dehydrated and cleaned with 95% ethyl alcohol, absolute ethyl alcohol, and xylene. The slides were mounted with resinous medium for image acquisition. For von Kossa staining, sections were incubated with 1% silver nitrite solution under a 60-W lamp for 1 hour. Slides were washed three times in distilled water and incubated with a 5% solution of sodium thiosulfate for 5 min. Slides were washed three times in distilled water and counterstained with 0.1% nuclear fast red. Slides were rinsed three times in distilled water and dehydrated before mounted in mounting medium. For H&E stain, rehydrated slides were incubated in Mayer’s Hematoxylin for 10 min, rinsed in running tap water for 10 min, incubated in Eosin for 30 s, washed in distilled water for 10 min and dehydrated as detailed above. Representative images were selected based on the mean values of histological scores.

### Skeletal preparation and μCT scanning analysis

The protocol for Alcian blue staining for cartilage and Alizarin red staining for mineralized tissue has been previously described [21]. µCT scanning of postnatal long bones was conducted using a SCANOCO µCT 50 according to standard procedures and data were analyzed using software from the manufacturer. Representative images were selected based on the mean values of μCT scores.

### RNA-seq

Total RNA was isolated from the shaft of the humerus of P0 neonatal after carefully removing skin, muscle, the ends and flushing out the bone marrow. Three wild types and three Prx1Cre; Ror2^flox/flox^ neonatal were used for total RNA isolation. RNA library construction, sequencing and analysis were provided by Azenta life science. The top GO categories were selected according to the P values.

### Statistics

All mice used in this study were randomly assigned to each group. Statistical analyses were completed using the Prism GraphPad. The Shapiro-Wilk test was used to normality test of the data. The Mann-Whitney U test was used for non-normally distributed data. Data are represented as average ± standard error of the mean (SEM) of at least three independent experiments. A two-way ANOVA was applied to analyze the three or more groups. Differences with *P*<0.05 were considered statistically significant.

## RESULTS

### Ror2 deficiency in early limb mesenchyme resulted in limb defects similar to those in RS patients

**Figure 1.**
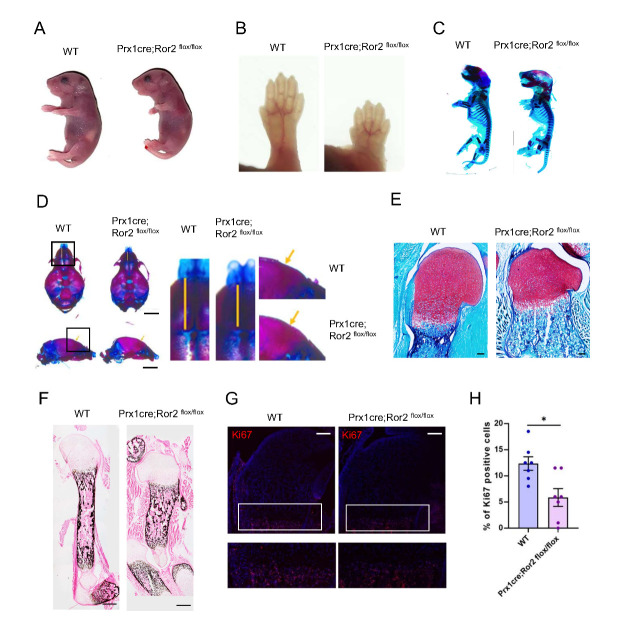
**Dysfunction of Ror2 in Prx1+ mesenchymal progenitors during embryonic development induces Robinow syndrome-like phenotypes**. (**A**) Gross appearance of the WT and CKO ^Prx1^ P0 mice. (**B**) Representative images of forelimb in WT and CKO ^Prx1^ P0 mice. (**C**) Whole-mount Alizarin red and Alcian blue staining of WT and CKO ^Prx1^ P0 mice. (**D**) Whole-mount Alizarin red and Alcian blue staining of the calvarium in WT and CKO ^Prx1^ P0 mice. Scale bar: 250m. (**E**) Safranin O staining of the humorous from P0 WT and CKO ^Prx1^. Scale bar: 500m. (**F**) Von kossa staining of the humorous from the P0 WT and CKO ^Prx1^. Scale bar:500m. (**G**) Immunostaining of Ki67 (red) and DAPI (blue) in the humorous from the P0 WT and CKO ^Prx1^. Scale bar:200m. (**H**) Quantification of percentage of Ki67-positive cells in G. Data were analyzed using the Mann-Whitney test and expressed as means ± SEM; n=5 per group; *P<0.05.

Ror2 plays important roles in the morphogenesis of various organs. To examine the cell of origin and the regulatory mechanisms regulating the skeletal phenotype in RS patients, Ror2 was deleted in early mesenchymal progenitor cells by crossing the Prx1cre line, in which cre is expressed in early limb bud mesenchyme, with conditional Ror2^flox/flox^ mice. The Prx1cre; Ror2^flox/flox^ pups were born at Mendelian ratios and could be distinguished from their wild-type littermates due to their shorter limbs (Fig. 1A). The Prx1cre; Ror2^flox/flox^ pups displayed marked shortening of the distal phalanges (Fig. 1B). Moreover, they exhibited short and thick long bones, as visualized byalizarin red/alcian blue staining of the skeleton for mineralized tissue and cartilage, respectively (Fig. 1C). These mutant mice survived postnatally with no remarkable differences in terms of body weight and length. Additionally, compared with their littermate controls, the conditional knockout mice showed shorter snouts and arched skulls (Fig. 1D), consistent with the expression pattern of Prx1cre in the craniofacial region. Histological analysis revealed alterations in the humerus cartilage of CKO newborns, such as a shortened growth plate (Fig. 1E) and decreased mineralization in the subchondral bone (Fig. 1F). Immunofluorescence analysis indicated reduced growth plate chondrocyte proliferation and a shorter proliferative zone in the Prx1cre; Ror2^flox/flox^ mutants (Fig. 1G, H). These findings suggest that is required for early mesenchymal limb bud development, and the dysfunction of Ror2 in mesenchymal lineage cells contributes to the skeletal abnormities observed in RS.

### Loss of Ror2 in osteoblast lineage cells impaired bone formation during adult skeletal development and promoted adipogenesis in the bone marrow in vivo

**Figure 2.**
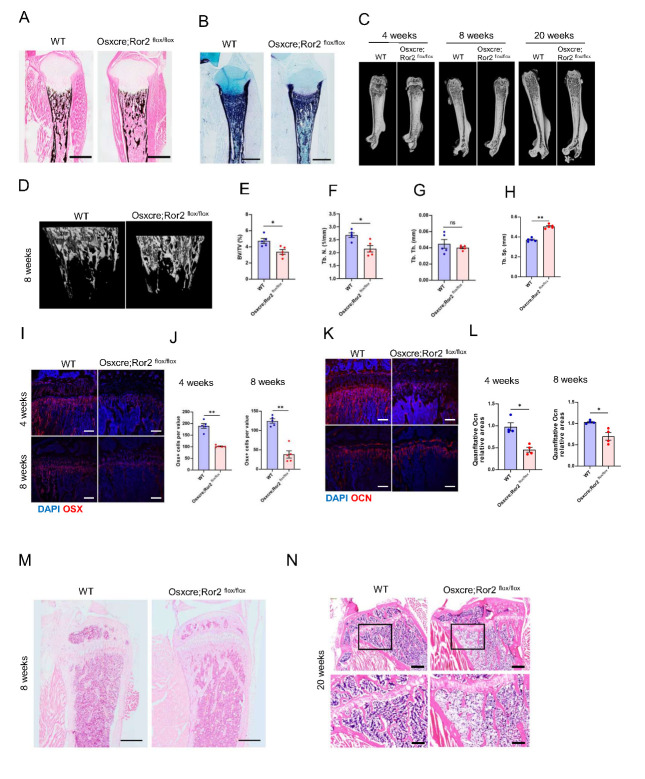
**Loss of Ror2 in Osx+ progenitors leads to skeletal dysplasia and increased bone marrow adipocytes**. (**A**) Von Kossa and (B) ALP staining of the tibia from P0 WT and CKO ^Osx^. Scale bar: 500m. (**C**) Three-dimensional -CT images of the femur from 4-week-old, 8-week-old, and 20-week-old WT and CKO ^Osx^ mice. (**D**) Three-dimensional -CT images of the trabecular bone in distal femurs isolated from 8-week-old WT and CKO^Osx^ mice. (**E**) -CT analysis of the distal femurs from 8-week-old WT and CKO ^Osx^ mice, showing trabecular bone volume per tissue volume (BV/TV), n=5 per group. (**F**) Trabecular number (Tb. N), n=5 per group. (**G**) Trabecular thickness (Tb. Th), n=5 per group. (**H**) Trabecular separation (Tb. Sp), n=5 per group. (E-H) Data were analyzed using the Mann-Whitney test and expressed as means ± SEM; ns, no significance, *P<0.05 and **P<0.01. (**I**) Immunostaining of Osx (red) and DAPI (blue) in the tibias from 4-week-old and 8-week-old WT and CKO ^Osx^ mice. Scale bar: 200m. (**J**) Quantification of the number of Osx+ cells per value in (I), n=4 per group, data were analyzed using the Mann-Whitney test and expressed as means ± SEM; **P<0.01. (**K**) Immunostaining of Ocn (red) and DAPI (blue) in the tibias from 4-week-old and 8-week-old WT and CKO ^Osx^ mice. Scale bar: 200 m. (**L**) Quantification of the relative area of Ocn in (K), n=4 per group, data were analyzed using the Mann-Whitney test and expressed as means ± SEM; *P<0.05. (**M**) H&E staining of the mesial tibia from 8-week-old WT and CKO^Osx^ mice. Scale bar: 500 m. (**N**) H&E staining of the mesial tibia from 20-week-old WT and CKO^Osx^ mice. Scale bar: 500 m.

To determine whether the RS-like phenotype in the Prx1cre; Ror2 ^floxflox^ mice resulted fromreduced osteoblast differentiation, we utilized the Osx::GFP-cre line to delete Ror2 in osteoblast lineage cells throughout theskeletal system. The Osxcre; Ror2^floxflox^ mice exhibited normal viability and fertility. Interestingly, these mice normal stature (Fig. 2A-C). Von Kossa staining of neonatal pups revealed decreased mineralization in the mutants compared to control mice (Fig. 2A). ALP staining at the equivalent age demonstrated a noticeable decrease in inosteoblast differentiation in the periosteum (Fig. 2B). As Osx is expressed in hypertrophy, perichondrium, and periosteum, while Ror2 is known to be expressed in the resting and proliferative zones of growth plate and perichondrium, but not the hypertrophic zone at postnatal day 3, these results indicate that skeletal defects in RS are not due to hypertrophy disrupting ossification.

To further investigate whether bone formation was affected by Osxcre-driven mutation, Micro-quantitative computed tomography (µCT) analysis was performed to compare the stature changes and bone-related elements in the long bones of CKO^Osx^ mice and their WT littermates. Three dimensional reconstructions of femurs from 4-week-old to 20-week-old mice revealed no obvious differences in terms of thickness and length (Fig. 2C). However, 8-week-old Osxcre; Ror2 ^floxflox^ mice exhibited a significant decrease in bone mass (Fig. 2D). Trabecular bone per tissue volume (BV/TV) in the CKO^Osx^ mice was reduced compared to the control group (Fig. 2E), consistent with a decrease in trabecular number (Tb.N) (Fig. 2F), a reduction in trabecular bone thickness (Tb. Th) and an increase in trabecular bone spacing (Tb. Sp) (Fig. 2G). Immunofluorescence staining in the mesial tibia of CKO^Osx^ mice revealed decreased expression of the osteoblast markers Osxterix (Osx) (Fig. 2I, J) and osteocalcin (Ocn) (Fig. 2K, L), indicating that decreased bone formation was due to weakened osteoblast differentiation. H&E staining results of CKO^Osx^ mouse tibia revealed increased adipocyte accumulation in the tibia bone marrow (Fig. 2M) and an increase in fat vacuoles in the bone marrow cavity in older CKO mice (Fig. 2N).

### Reduced endochondral ossification with delayed chondrocyte differentiation in mesenchymal progenitor cells

**Figure 3.**
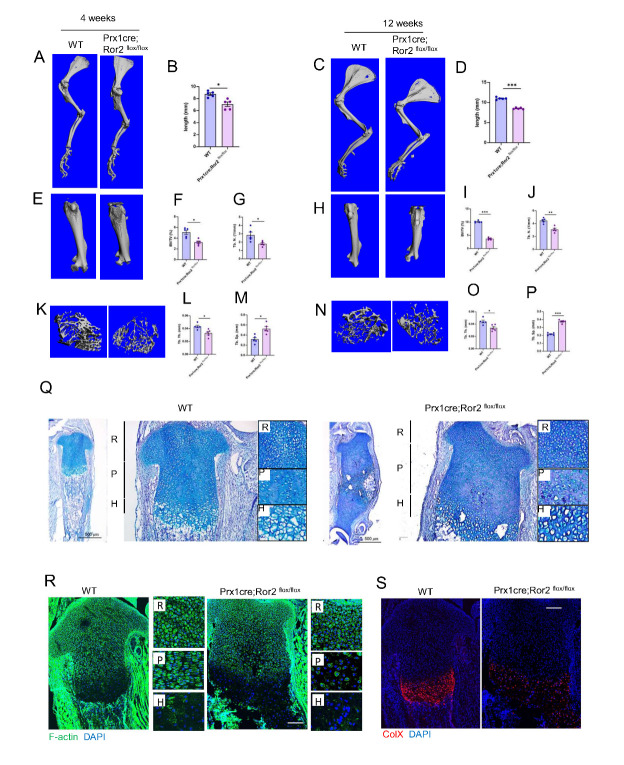
**Ror2 is required for cartilage development and chondrocyte organization**. (**A**) Representative -CT image of the humerus from 4-week-old WT and CKO ^Prx1^ mice. Scale bar: 5 mm. (**B**) Quantification of humerus length in (A), n=5. (**C**) Representative -CT image of the humerus from 12-week-old WT and CKO ^Prx1^ mice. Scale bar: 5 mm. (**D**) Quantification of humerus length in (C), n=5. (**E**) Representative -CT image of the femur from 4-week-old WT and CKO ^Prx1^ mice. (**F**) -CT analysis of distal femurs from 4-week-old WT and CKO ^Prx1^ mice, n=5, showing trabecular bone volume per tissue volume (BV/TV); (G) Trabecular number (Tb. N); n=5. (**L**) Trabecular thickness (Tb. Th), n=5. (**M**) Trabecular separation (Tb. Sp), n=5. (**H**) Representative -CT image of the femur from 12-week-old WT and CKO ^Prx1^ mice. (**I**) -CT analysis of distal femurs from 12-week-old WT and CKO ^Prx1^ mice, n=5, showing trabecular bone volume per tissue volume (BV/TV). (**J**) Trabecular number (Tb. N); n=5. (**O**) Trabecular thickness (Tb. Th). (**P**) Trabecular separation (Tb. Sp). Data were analyzed using the Mann-Whitney test and expressed as means ± SEM. *p<0.05, **p<0.01 and ***P<0.001. (**K**) Three-dimensional -CT images of the trabecular bone in distal femurs isolated from 8-week-old WT and CKO ^Prx1^ mice. (**N**) Three-dimensional -CT images of trabecular bone in distal femurs isolated from 12-week-old WT and CKO ^Prx1^ mice. (**Q**) Alcian blue staining and (R) Phalloidin staining of the tibia from P0 WT and CKO ^Prx1^ mice, showing Resting (R), proliferative (P), and hypertrophic (H) areas. (**S**) Immunostaining of ColX (red) and DAPI (blue) in the tibias from P0 WT and CKO ^Osx^ mice. Scale bar: 150 m.

Given previous studies indicating a significant clonality switch in growth plate chondrocytes shortly after birth, we investigated the role of Ror2 in mesenchyme for regulating endochondral ossification and chondrocyte differentiation during postanal skeletal development. The Prx1cre; Ror2 ^flox/flox^ mice and their age-matched controls were subjected to µ-CT analysis to compare the length and bone-related elements of long bones. Three-dimensional reconstructions of humerus and femur in 4-week-old to 12-week-old CKO^Prx1^ mice revealed a significant decrease in the length compared to controls (Fig. 3A-D). Quantitative analysis of the distal femur showed a significant decrease in bone mass (Fig. 3K, N) in the CKO^Prx1^ mice compared to WT littermates at identical postnatal age, consistent with a decrease in trabecular number (Tb. N), trabecular thickness (Tb. Th), and an increase in trabecular bone spacing (Tb. Sp) (Fig. 3F-P). Although Prx1cre induces more complete deletion in forelimbs, µ-CT reconstructions revealed a modest reduction in hindlimb length in Ror2 CKO mice (Fig. 3E, H). Histological analyses of late embryonic stage tibias revealed significant alterations in the tibia growth plate of Ror2 CKO newborns, notably an enlarged proliferating zone and delayed hypertrophy (Fig. 3Q). To investigate if the cartilage abnormalities arise from defects in the polarity, we used phalloidin to visualize the cytoskeleton of chondrocytes. The results demonstrated disrupted chondrocyte polarity and organization in the proliferating zone of Ror2 CKO mice (Fig. 3R). Immunofluorescence of mature chondrocyte marker collagen type 10a1 identified stalled chondrocyte maturation prior to terminal ossification (Fig. 3S). Ror2 is known to be expressed in reserve and proliferating chondrocytes in older embryos, and loss of the receptor in mesenchymal progenitor cells impaired the initial growth and pattern of developing cartilage anlagen and their growth plate. It continues to be required for the normal polarity of chondrocytes from the proliferative to the hypertrophic zone.

### Ror2 is required for BMP/TGF-β signaling in the developing growth plate

**Figure 4.**
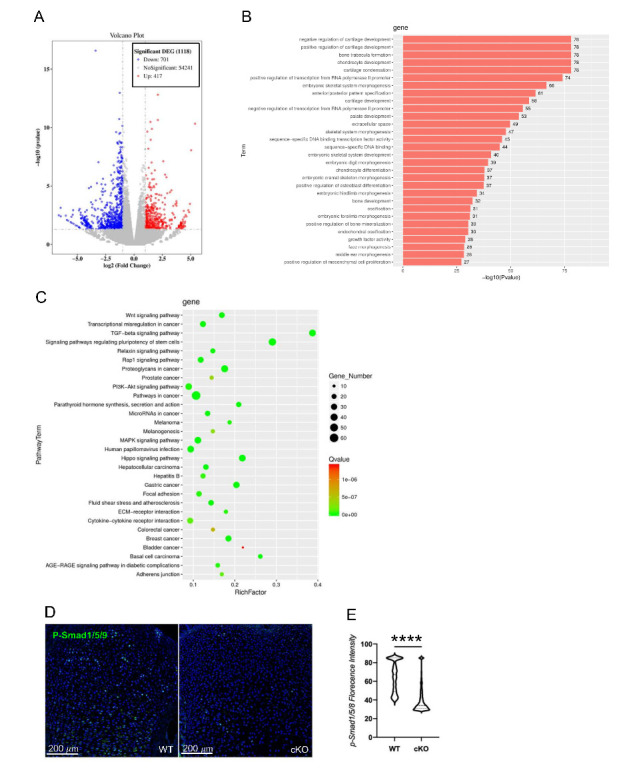
**Deletion of Ror2 leads to a reduction in BMP/TGF- signaling pathway activity in developing long bones**. (**A**) Volcano plot analysis of bulk RNA- seq data comparing WT and CKO ^Prx1^ P0 mice. (**B**) GO enrichment analysis of the differentially expressed genes between WT and CKO ^Prx1^ P0 mice. (**C**) KEGG analysis showing the enrichment of all genes in bulk RNA- seq. (**D**) Immunostaining of p-smad1/5/8 (green) and DAPI (blue) in the tibias from P0 WT and CKO ^Osx^ mice. Scale bar: 200 m. (**E**) Quantification of p-smad1/5/8 + cells in (D). n=6, Data were analyzed using the Mann-Whitney test and expressed as means ± SEM, P<0.0001.

To investigate the molecular mechanism underlying the regulation of chondrocyte differentiation by Ror2, we conducted bulk RNA sequencing analysis using developing humerus from the neonatal pups, we profiled profound gene expression changes (Fig. 4A). Gene ontology (GO) enrichment analysis for biological processes revealed significant alterations in the cartilage developmental process (Fig. 4B). Notably, genes associated with ossification, skeletal development, and bone development showed significantly downregulated expression (Fig. 4B). Further genome pathway analysis highlighted significant changes in several signaling pathways, including Wnt signaling, TGF-β signaling, and Hippo signaling. Among these pathways, the TGF-β signaling pathway exhibited asignificant reduction in the Ror2 CKO^Prx1^ mouse (Fig. 4C). To validate these findings, immunohistochemistry was performed on newborn tibia sections, demonstrating robust staining for canonical downstream BMP signaling activators, p-SMAD1/5/8, primarily localized within the proliferative zone of growth plate (Fig. 4D, E) in the control mice. In contrast, the Ror2 mutant mice exhibited moderate p-SMAD1/5/8 staining throughout the growth plate. Given the crucial role of BMP/TGF-β signaling in cartilage development and patterning, these results suggest that Ror2 may regulate chondrocyte differentiation through its interaction with the BMP/TGF-β signaling pathway.

### Upregulated BMP/TGF-β signaling partially rescues skeletal abnormalities in Ror2 mutant mice

**Figure 5.**
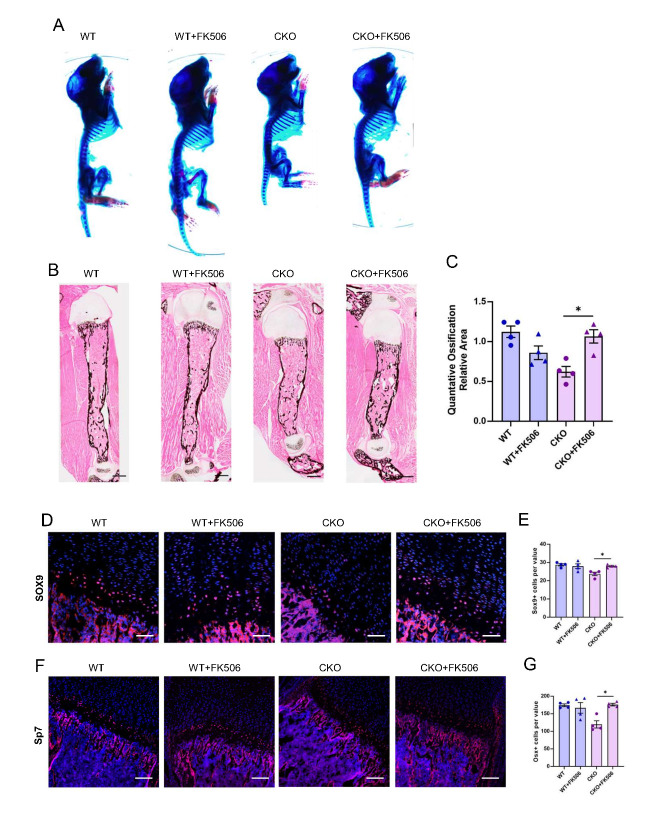
**Profound skeletal abnormalities in CKO ^Prx1^ mice were partially rescued by FK506 treatment**. (**A**) Whole-mount Alizarin red and Alcian blue staining of littermate mice of indicated genotype at P7. The forelimb (FL) and hindlimb (HL) are shown in the lower panel. (**B**) Von Kossa staining of the tibia from P7 WT and CKO ^Prx1^. Scale bar: 500 m. (**C**) Quantification of ossification relative area in C, n=4 per group. (**D**) Immunostaining of Sox9 (red) and DAPI (blue) in the tibias from P7 WT and CKO ^Prx1^ mice. Scale bar: 150 m. (**E**) Quantification of Sox9+ cells per value in D, n=4 per group. (**F**) Immunostaining of Osx (red) and DAPI (blue) in the tibias from P7 WT and CKO ^Prx1^ mice. Scale bar: 150 m. (**G**) Quantification of Osx+ cells per value in F, n=4 per group. (C-G) Data were analyzed using the two-way ANOVA and expressed as means ± SEM, *P<0.05.

To investigate whether increased BMP/TGF-β signaling can rescue the skeletal abnormalities and chondrocyte differentiation defects observed in Ror2 mutant mice, we treated neonatal pups and their control littermateswith FK506, FK506is known to upregulate BMP signaling by dissociating intracellular BMP repressor FKBP12 from BMP receptor-1 (BMPR1) [22]. We observed substantial rescue of the skeletal phenotypes and chondrocyte differentiation in the Ror2 CKO Prx1 mice after FK506 treatment during the early postnatal stage (Fig. 5A). The restoration of short stature was visualized through whole-mount skeletal preparation analysis. Von Kossa staining of the humerus from treated mice showed a significant increase in ossification areas when compared to untreated mice (Fig. 5B, C).

At the molecular level, FK506 administration resulted in a significant upregulation of Sox9 and Osx expression (Fig. 5D to G), which are key factors involved in the regulation of chondrocytes and pre-osteoblast differentiation. These findings indicate that Ror2 deficiency in mesenchymal stem cells disrupts chondrocyte polarity and bone formation by reducing BMP/TGF-β signaling. Consequently, targeting BMP/ TGF-β signaling could be a potential therapeutic approach to improve skeletal development in patients with RS.

## DISCUSSION

Mutations in the ROR2 gene in humans have been identified as the cause of recessive RS, which is characterized by severe skeletal dysplasia, including generalized limb bone shortening, segmental spine defects, brachydactyly, and distinctive facial features [23, 24]. In this study, we demonstrate that deleting Ror2 function in early embryonic mesenchymal stem cells leads to skeletal malformations and disorganized growth plate pattern that resembles the phenotypes observed in RS. On the other hand, when Ror2 is removed from osteoblast lineage cells, it results in reduced bone formation and increased adipogenesis, but but without affecting stature. These findings provide further insight into the role of Ror2 in skeletal development and the pathogenesis of Robinow syndromeRS.

Indeed, we identified abnormal craniofacial development in our Prx1 driven Ror2 conditional knock out mice such as shorter snouts and arched skull visualized by skeletal preparation (Fig. 1D). Whereas in human, it was reported that gingival hyperplasia, orbital hyperplasia, a short nose with anteverted and flared nares, a triangular mouth with a long philtrum, cleft palate, macrocephaly, and frontal bossing could both affect dominant Robinow syndrome and (DRS) and recessive Robinow syndrome (RRS) [25]. Previous studies reported the cause of RRS is due to ROR2 mutation, typical face with everted nostrils, large mouth and hypertelorism were listed as phenotype of human RRS [8]. Besides, some RRS patients also have dental problems, such as fusion of primary teeth, delayed eruption of the permanent teeth and delayed root formation of the permanent teeth [26]. Ror2-/- mice exhibited retarded crown formation and defective odontoblast differentiation [27]. Furthermore, loss of Ror2 in the dental mesenchyme driven by Osr2-Cre leads to delated root elongation and ultimately shortened roots [28].

Ror2 has been demonstrated to have critical roles in osteoblast differentiation and cartilage response to inflammatory cytokines and mechanical stress [17, 29]. In vitro studies have shown that overexpression of Ror2 inhibits chondrocyte differentiation, while blocking its expression promotes chondrocyte differentiation and suppresses the expression of the cartilage-degrading enzymes in C3H10t1/2 cells [30]. Therefore, the skeletal abnormalities observed in RS are likely attributed to the malfunction of Ror2 in mesenchymal lineage cells during both embryonic and juvenile development stages, affecting morphogenetic information formation. In our study, we identified the induction of BMP/TGF-β signaling as a crucial function of Ror2 during pattern formation. Pharmacological enhancement ofBMP/TGF-β signaling partially rescued the bone phenotypes of the Ror2 loss-of-function mutants, suggesting that modulation this pathway holds promise as a potential approach to ameliorate the skeletal phenotypes in RS.

Canonical Wnt signaling has been shown to play a supportive role in the regulation of bone formation by osteoblasts [31-36]. In contrast, activation of noncanonical Wnt/Ror2 signaling tends to favor bone resorption [37]. Studies have reported in Wnt5a+/- and Ror2^+/-^ transgenic mice, the Wnt5a-Ror2 cascade between osteoblast and osteoclast precursors promotes osteoclstogenesis [12]. Our findings further demonstrate that Ror2 has a more pronounced effect in mesenchymal progenitor cells compared to differentiating osteoblasts. Deletion of Ror2 in mesenchymal progenitors of cartilage and bone leads to significantly more severe skeletal dysplasia compared to Ror2 loss in Osx-expressing cells. Specifically, no defects in cartilage formation or chondrocyte differentiation were observed in Osxcre;Ror2^flox/flox^ embryos, but decreased osteogenic differentiation and adipogenesis in the bone marrow were observed in adulthood. Further investigations could explore whether Ror2 also contributes to this fate change.

BMP/TGF-β signaling plays a crucial role in the normal development of cartilage-derived bone tissue in both mouse and human [38, 39]. -Malfunction of BMP/ TGF-β signaling have been linked to various human skeletal dysplasias. Brachydactyly type A2, for instance, results from loss-of-function mutations in, which encodes the type I BMP receptor ALK6, and BMP2 [40, 41]. Mutations in the BMP antagonist Noggin (NOG) and ROR2 cause Brachydactyly type B2 (BDB2) [4]. Noggin has been shown to enhance theactivation of the Wnt-5a-Ror2-Disheveled (Dvl) pathway in mouse embryonic fibroblast (MEF) cells, depending on Ror2 [42]. Additionally, a functional and physical association between Ror2 and BMP receptor type Ib (BRIb) has been observed,, with the Ror2/ BRIb complex forming in distinct microdomains at the plasma membrane [43, 44]. Through bulk RNA sequencing, we have demonstrated decreased BMP/ TGF-β signaling activity in Prx1cre; Ror2^flox/flox^ mutant mice during the embryonic stage. Notably, treatment with FK506 partially alleviates skeletal defects at this stage.

Tacrolimus (FK506) could bind to FK506 binding protein (FKBP) to form a complex. It was recognized that BMP pathway inhibitors FKBP12 and casein kinase 2 endogenously limit the activities of the type one receptors and may be inactivated by delivery of FK506 to increase BMP signal transduction [45]. Tacrolimus can inhibit calcineurin phosphatase, which inhibits T-lymphocyte signal transduction and IL-2 transcription to achieve immunosuppressive properties [46, 47]. The small molecule FK506 is widely known as a FDA approved immunosuppressant, and it has been reported to inhibit the mice glomerular mesangial cell proliferation by affecting the TGF-β and Smads signal [48]. Furthermore, the FK506 was proved to activate BMPR2 and rescued endothelial dysfunction and reverses pulmonary hypertension [22]. Indeed, the systematic small molecule delivery may not only target the bona cells. Therefore, future experiments such as genetic overexpression using siRNA or conditional mouse models need be explored to understand the inherent mechanisms.

Collectively, our studies provide insights into the cellular origin (mesenchymal progenitors) and molecular mechanism (BMP/TGF-β signaling) underlying the skeletal dysplasia by modeling the phenotype of RS in mice.
